# Nicotinamide Riboside Alleviates Cardiac Dysfunction and Remodeling in Pressure Overload Cardiac Hypertrophy

**DOI:** 10.1155/2021/5546867

**Published:** 2021-09-15

**Authors:** Sai Ma, Jing Feng, Xiuyu Lin, Jing Liu, Yi Tang, Shinan Nie, Jianbin Gong, Lei Wang

**Affiliations:** ^1^Department of Cardiology, Jinling Hospital, Medical School of Nanjing University, Nanjing, China 210002; ^2^Department of Emergency Medicine, Jinling Hospital, Medical School of Nanjing University, Nanjing, China 210002; ^3^Department of Ultrasound Diagnostic, Jinling Hospital, Medical School of Nanjing University, Nanjing, China 210002

## Abstract

**Background:**

Cardiac hypertrophy is a compensatory response to pressure overload, which eventually leads to heart failure. The current study explored the protective effect of nicotinamide riboside (NR), a NAD^+^ booster that may be administered through the diet, on the occurrence of myocardial hypertrophy and revealed details of its underlying mechanism.

**Methods:**

Transverse aortic constriction (TAC) surgery was performed to establish a murine model of myocardial hypertrophy. Mice were randomly divided into four groups: sham, TAC, sham+NR, and TAC+NR. NR treatment was given daily by oral gavage. Cardiac structure and function were assessed using small animal echocardiography. Mitochondrial oxidative stress was evaluated by dihydroethidium (DHE) staining, malondialdehyde (MDA) content, and superoxide dismutase (SOD) activity. Levels of expression of atrial natriuretic peptide (ANP), brain natriuretic peptide (BNP), IL-1*β*, TNF-*α*, and Sirtuin3 were measured by real-time PCR and ELISA. Expression levels of Caspase-1, Caspase-1 pro, cleaved Gasdermin D (GSDMD), NLRP3, ASC, Sirtuin3, ac-MnSOD, and total MnSOD were measured by Western blot.

**Results:**

Reductions in the heart/body mass ratio (HW/BW) and lung/body mass ratio (LW/BW) and in ANP, BNP, and LDH levels were observed in the TAC group on the administration of NR (*P* < 0.05). Moreover, echocardiography data showed that cardiac dysfunction and structural changes caused by TAC were improved by NR treatment (*P* < 0.05). NR treatment also reduced levels of the inflammatory cytokines, IL-1*β* and TNF-*α*, and attenuated activation of NLRP3 inflammasomes induced by TAC. Furthermore, changes in DHE staining, MDA content, and SOD activity indicated that NR treatment alleviated the oxidative stress caused by TAC. Data from ELISA and Western blots revealed elevated myocardial NAD^+^ content and Sirtuin3 activity and decreased acetylation of MnSOD after NR treatment, exposing aspects of the underlying signaling pathway.

**Conclusion:**

NR treatment alleviated TAC-induced pathological cardiac hypertrophy and dysfunction. Mechanically, these beneficial effects were attributed to the inhibition of NLRP3 inflammasome activation and myocardial inflammatory response by regulating the NAD^+^-Sirtuin3-MnSOD signaling pathway.

## 1. Introduction

Cardiac hypertrophy is a feature common to many cardiovascular diseases [1]. Hypertrophy develops as a benign, adaptive response to physiological and pathological stimuli and results in increased heart contractility. However, with continued stimulation, hypertrophy progresses to cardiac remodeling and dysfunction and becomes pathological hypertrophy, a complex process which eventually leads to heart failure [2]. Recent research has identified a number of mechanisms that contribute to pathological hypertrophy, including cellular metabolism, the immune response, abnormal expression of lncRNAs, and epigenetic changes [3, 4]. However, there still remains a need to conduct further exploration in order to identify molecular targets for treatment.

Inflammation is considered a critical hallmark of hypertrophy [5]. However, the pathogenic role of inflammation during hypertrophy is incompletely understood. The cytosolic inflammasome is a multiprotein complex, comprising key regulators of the inflammatory response to numerous stimuli. The resulting activation of Caspase-1 induces maturation of proinflammatory cytokines [6]. There is increasing evidence that links the nucleotide-binding and leucine-rich repeat pyrin domains protein 3 (NLRP3) inflammasome and its stimulation of cytokine secretion with the pathogenesis of various cardiovascular diseases, including atherosclerosis, acute myocardial infarction (AMI), acute myocarditis, and progression to heart failure (HF). Such findings expose the possibility of oral administration of NLRP3 inflammasome inhibitors to treat cardiovascular disorders [7]. Hence, there is increasing scrutiny of inflammasome-based approaches to cardiovascular treatment.

Nicotinamide adenine dinucleotide (NAD^+^) is a coenzyme involved in diverse biological processes [8]. Decreased NAD^+^ levels and subsequent mitochondrial protein hyperacetylation have been reported to be associated with the development of HF. Thus, there is the potential for elevating NAD^+^ levels during therapy for cardiovascular diseases [9]. Notably, the potential benefits of dietary administration of NAD^+^ boosters have attracted much attention [10]. However, the salubrious effects of such agents in the hypertrophic heart have not been explored.

In the present study, we have used a transverse aortic constriction (TAC) mouse model to demonstrate that NLRP3 inflammasome activation is associated with the progression of heart hypertrophy. Furthermore, we have identified a cardioprotective role for nicotinamide riboside (NR), an NAD^+^ booster available through the diet and which inhibits the NLRP3 inflammasome and inflammatory responses via regulation of the Sirtuin3-MnSOD signaling pathway.

## 2. Materials and Methods

### 2.1. Materials

#### 2.1.1. Animals

Male C57BL/6J mice (8 weeks, 180-200 g weight) were purchased from the Animal Center of Jinling Hospital. Animals were maintained under standard conditions of temperature (21 ± 2°C) and humidity (55 ± 2%) with an alternating 12 h light/12 h dark cycle. The animals had free access to tap water and were fed adequate food. The procedures of *in vivo* study were approved by the Jinling Hospital Committee on Animal Care. All animal study procedures were carried out in accordance with the Chinese National Institutes of Health Guidelines on the Use of Laboratory Animals.

#### 2.1.2. Reagents

NR was purchased from China Thompson Biological Co., Ltd. DHE probe was purchased from Biolab company (Frozen Section ROS Detection Kit, Biolab, Beijing, China). Antibodies raised against Caspase-1, Caspase-1 pro, NLRP3, ASC, Sirtuin3, and *β*-actin for Western blotting were purchased from Cell Signaling Technology (Cell Signaling Technology, USA), and antibodies raised against cleaved GSDMD, MnSOD (total), and ac-MnSOD (MnSOD-acetyl K68) were purchased from Abcam (Abcam, USA).

### 2.2. Methods

#### 2.2.1. Experimental Grouping and Surgical Treatments

A mouse model of cardiac hypertrophy was established using TAC surgery. A total of 40 experimental mice were randomly assigned to one of four groups: sham, TAC, sham+NR and TAC+NR with 10 mice in each group. The TAC procedure is as follows. Mice were anesthetized with 2% isoflurane. Under aseptic conditions, with the mouse supine, the second rib on the left side of the thoracic cavity was cut off with surgical scissors and the thymus lightly plucked to fully expose the aortic arch. A 27G pillow was placed along the direction of the aortic arch, followed by ligation with a 7-0 thin thread to constrict the aortic arch to about 0.4 mm in diameter when the pillow was removed. Mice in the sham group were subjected to skin incisions and blunt separation without constriction of the aorta. The chest cavity was closed and the skin disinfected with iodophor. Postsurgery, penicillin was injected intraperitoneally to prevent infection. Echocardiography was performed one week after TAC surgery to confirm the position of the ligation thread. Mice in the sham+NR and TAC+NR groups were given NR by daily oral gavage at a dose of 400 mg/kg/d for 8 weeks.

#### 2.2.2. Tissue Collection and Measurement

Eight weeks postsurgery, mice were fasted for 12 hours, weighed, and anesthetized by intraperitoneal injection of 2% sodium pentobarbital. Blood was taken from the carotid artery, and the heart and lungs were removed and weighed. The heart/body mass ratio (HW/BW): heart mass (mg)/body mass (g) and lung/body mass ratio (LW/BW): lung mass (mg)/body mass (g) were calculated. After standing for 15 minutes, carotid blood was centrifuged at 2,000g for 10 min to harvest serum. The activity of the myocardial injury marker, lactate dehydrogenase (LDH), was measured spectrophotometrically using a commercially available kit (Jiancheng Bioengineering Institute, Nanjing, China). Levels of IL-1*β* and TNF-*α* in myocardial tissue were measured using ELISA kits according to the manufacturer's instructions.

#### 2.2.3. Evaluation of Heart Function by Echocardiography

A Vevo 2100 ultrasound echocardiography system (Visual-Sonics, Toronto, Canada) was used to evaluate cardiac function. In brief, mice were anesthetized with isoflurane throughout. The heart was imaged in the two-dimensional parasternal short-axis view and an M-mode echocardiogram of the midventricle produced. Detailed procedures are as reported previously [11].

#### 2.2.4. Measurement of Myocardial Oxidative Stress

Dihydroethidium (DHE) staining was used to evaluate the production of myocardial reactive oxygen species (ROS) in response to TAC. Briefly, frozen tissue sections were incubated with DHE solution for 30 min in the dark at 37°C. Cell nuclei were counterstained by DAPI (1 mg/mL) for 5 min. After washing, the slides were visualized under a laser scanning confocal microscope (Olympus FV1200, Olympus, Tokyo, Japan). Three replicate sections were analyzed to calculate the mean data.

Levels of malondialdehyde (MDA) and activities of superoxide dismutase (SOD) in myocardial tissue were measured with commercial assay kits (Beyotime, Shanghai, China), according to the manufacturer's instructions.

#### 2.2.5. ELISA Assay

Levels of inflammatory cytokines, IL-1*β* and TNF-*α*, were measured in myocardial tissue using commercial ELISA kits (IL-1*β* and TNF-*α* ELISA kit: abs520001, Abison Shanghai Biotechnology Co., Ltd., China), according to the manufacturer's instructions. Each sample was tested in triplicate.

#### 2.2.6. Sirtuin3 Activity

The Sirtuin3 activity was analyzed using a commercial kit (Sirtuin3 Activity Assay Kit, Abcam, Cambridge, MA, USA), according to the manufacturer's instructions. Fluorescence at Ex/Em = 340–360/440–460 nm was measured using a microplate reader. Sirtuin3 activities are presented relative to the sham group.

#### 2.2.7. Quantitative Real-Time PCR

Quantitative real-time PCR was performed as described previously [11]. Briefly, total RNA was isolated from myocardial tissue, and cDNA was synthesized using a QuantiTect reverse transcription kit (Qiagen, Hiden, Germany). A real-time PCR procedure was performed using the KAPA SYBR FAST qPCR Kit (KAPA Biosystems, Woburn, MA, USA). Primer sequences are listed in Table 1. Forty cycles of amplification for genes encoding ANP and BNP were carried out (94°C for 30 s, 60°C for 60 s, and 72°C for 1 min). Relative mRNA expressions were calculated by the *ΔΔ*CT method using the 7500 System SDS Software Version 1.2.1.22 (Applied Biosystems). GAPDH (housekeeping gene) was included as an internal standard.

#### 2.2.8. Western Blotting

In brief, heart tissue was collected into Eppendorf tubes. Proteins were extracted by homogenizing tissue in RIPA buffer (Thermo RIPA buffer, Thermo Fisher Scientific, Waltham, MA) containing 1% Thermo Scientific Halt Protease Inhibitor Cocktail. Protein quantification was performed using the Thermo Scientific Pierce BCA Protein Assay Kit (Thermo Fisher Scientific, Waltham, MA). For the Western blot assay, the tissue homogenate was loaded onto SDS-PAGE gels; bands were transferred to the NC membrane (Millipore, Billerica, MA) and blocked with 5% adipoprotein, followed by incubation with the primary antibody at 4°C overnight (Caspase-1, Caspase-1 pro, NLRP3, ASC, GSDMD, Sirtuin3, MnSOD, MnSOD-acetyl K68, and *β*-actin (1 : 2000)). Membranes were washed with TBST solution and incubated with corresponding secondary antibody for 1 h at room temperature. Bands were visualized by a chemiluminescence detection kit (Thermo Electron Corp, Rockford, IL) and analyzed with ImageJ software (NIH ImageJ System, Bethesda, MD).

#### 2.2.9. Statistics

All data were statistically analyzed with Prism 6.0 software. The experimental data are expressed as the mean ± standard deviation (mean ± SD). Data comparison was performed by one-way ANOVA analysis, followed by Tukey's analysis. *P* < 0.05 is considered to be the threshold for statistical significance.

## 3. Results

### 3.1. NR Attenuated the Myocardial Hypertrophy Markers in TAC Mice

**Figure 1:**
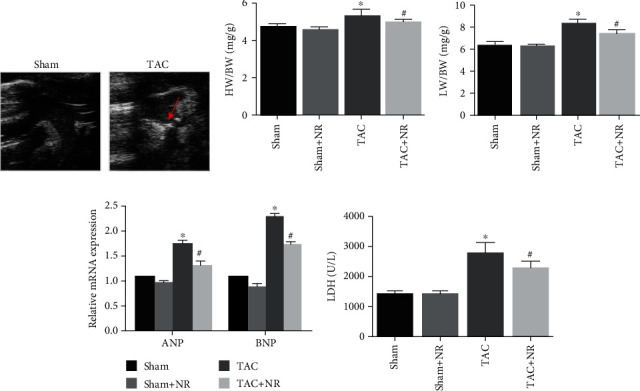
NR attenuates the myocardial hypertrophy markers in TAC mice. (a) Narrowed aorta (red arrow) in the TAC group. (b) The HW/BW ratio in the TAC+NR group was significantly reduced compared with the TAC group (5.011 ± 0.109 vs. 5.342 ± 0.323, *P* < 0.05). (c) NR reduced TAC-induced LW/BW ratio augmentation (7.544 ± 0.332 vs. 8.397 ± 0.320, *P* < 0.05). (d) NR significantly reduced ANP and BNP in TAC mice (ANP: 1.32 ± 0.08 vs. 1.76 ± 0.06, *P* < 0.05; BNP: 1.74 ± 0.05 vs. 2.31 ± 0.06, *P* < 0.05). (e) NR treatment reduced LDH level in hypertrophied mice (2300 ± 221 vs. 2978 ± 332 U/L, *P* < 0.05). TAC: transverse aortic constriction; HW/BW: heart/body mass ratio; LW/BW: lung/body mass ratio. ^∗^*P* < 0.05 vs. sham group; ^#^*P* < 0.05 vs. TAC group.

The results of ultrasound echocardiography, performed one week after TAC, are shown in Figure 1(a). The aorta was narrowed in the TAC group compared with the sham group. The effects of NR treatment on markers of myocardial hypertrophy, including cardiac mass index, ANP and BNP level, and LDH activity, were evaluated 8 weeks after TAC surgery. As shown in Figures 1(b) and 1(c), the HW/BW and LW/BW ratios in the TAC group (HW/BW: 5.342 ± 0.323 and LW/BW: 8.397 ± 0.320) were significantly higher than that in the sham group (4.771 ± 0.122; *P* < 0.05 and 6.426 ± 0.253; *P* < 0.05). NR treatment significantly reduced the ratios. The TAC+NR group had HW/BW: 5.011 ± 0.109 and LW/BW: 7.544 ± 0.332 compared with 5.342 ± 0.323 and 8.397 ± 0.320, respectively, for the TAC group (*P* < 0.05). Levels of ANP and BNP were lower in the NR+TAC group compared with the TAC group (ANP: 1.32 ± 0.08 vs. 1.76 ± 0.06; *P* < 0.05; BNP: 1.74 ± 0.05 vs. 2.31 ± 0.06; *P* < 0.05), indicating that NR treatment significantly improved the myocardial hypertrophy induced by TAC (Figure 1(d)). TAC caused an increase in activities of the cardiac injury marker, LDH, compared with the sham group (TAC: 2978 ± 332 U/L vs. sham: 1268 ± 219 U/L; *P* < 0.05), and activities were reduced on treatment with NR (TAC+NR: 2300 ± 221 U/L; *P* < 0.05) (Figure 1(e)).

### 3.2. NR Alleviated the Cardiac Dysfunction Caused by TAC

**Figure 2:**
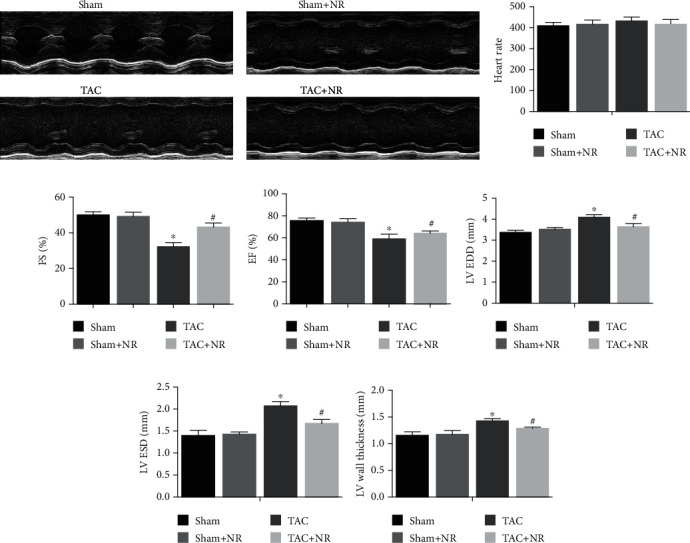
NR significantly alleviates the cardiac dysfunction caused by TAC. (a) The M-mode echocardiograms of the midventricle at the level of the papillary muscles. (b) There was no significant difference in mouse heart rate (HR) among groups (*P* > 0.05). (c, d) NR treatment significantly improved the left ventricular systolic function of TAC mice (EF: 64.3 ± 2.1 vs. 59.2 ± 4.2; FS: 43.2 ± 2.3 vs. 32.3 ± 2.2, *P* < 0.05). (e–g) NR treatment significantly improved TAC-induced changes in cardiac structure (LV EDD: 3.644 ± 0.154 vs. 4.096 ± 0.122; LV ESD: 1.676 ± 0.087 vs. 2.071 ± 0.096; LV wall thickness: 1.288 ± 0.023 ± 1.428 ± 0.040, *P* < 0.05). HR: heart rate; EF: ejection fraction; FS: fractional shortening; LV EDD: left ventricular end-diastolic dimension; LV ESD: left ventricular end-systolic dimension. ^∗^*P* < 0.05 vs. sham group; ^#^*P* < 0.05 vs. TAC group.

Heart structure and function were evaluated by ultrasound echocardiography.  Figure 2(a) shows the M-mode echocardiograms of the midventricle at the level of the papillary muscles. There was no significant difference in the mouse heart rate (HR) among groups with the HR ranging from 400 to 450 bpm (Figure 2(b)). The ejection fraction (EF) and fractional shortening (FS) were markedly decreased in the TAC group (EF: TAC: 59.2 ± 4.2 vs. sham: 75.8 ± 2.3; FS: TAC: 32.3 ± 2.2 vs. sham: 50.1 ± 1.6; *P* < 0.05), suggesting impaired myocardial function induced by TAC. NR treatment restored the left ventricular systolic function of TAC mice (EF: TAC+NR: 64.3 ± 2.1 vs. TAC: 59.2 ± 4.2; FS: TAC+NR: 43.2 ± 2.3 vs. TAC: 32.3 ± 2.2; *P* < 0.05) (Figures 2(c) and 2(d)). As is shown in Figures 2(e)–2(g), TAC caused changes in the structure of the mouse heart, manifested as left ventricular end-diastolic dimension (LV EDD), left ventricular end-systolic dimension (LV ESD), and left ventricular wall thickness (LV wall thickness) increases (LV EDD: TAC: 4.096 ± 0.122 vs. sham: 3.379 ± 0.092; LV ESD: TAC: 2.071 ± 0.096 vs. sham: 1.400 ± 0.115; LV wall thickness: TAC: 1.428 ± 0.040 vs. sham: 1.156 ± 0.066; *P* < 0.05). NR treatment significantly improved TAC-induced changes in cardiac structure (LV EDD: TAC+NR: 3.644 ± 0.154 vs. TAC: 4.096 ± 0.122; LV ESD: TAC+NR: 1.676 ± 0.087 vs. TAC: 2.071 ± 0.096; LV wall thickness: TAC+NR: 1.288 ± 0.023 vs. TAC: 1.428 ± 0.040; *P* < 0.05).

### 3.3. NR Inhibits the NLRP3 Inflammasome Activation and Inflammatory Cytokine Expression Induced by TAC

**Figure 3:**
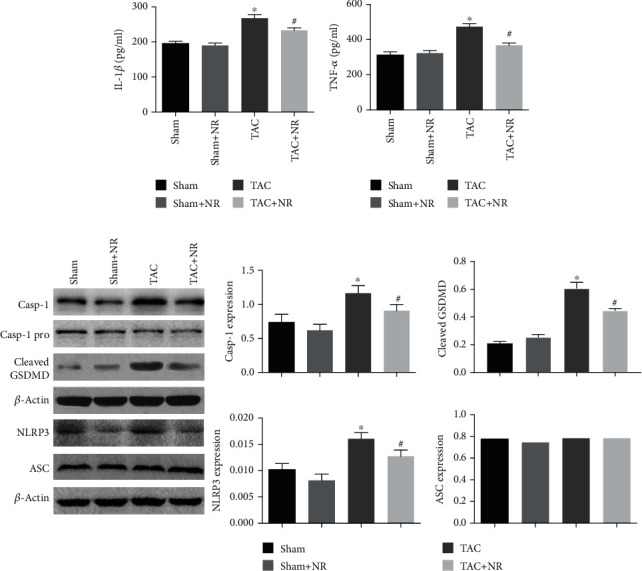
NR inhibits NLRP3 inflammasome activation induced by TAC. (a, b) NR significantly reduced the levels of high inflammatory factors caused by myocardial hypertrophy (IL-1*β*: 233.17 ± 7.12 vs. 267.58 ± 10.23; TNF-*α*: 366.9 ± 13.40 vs. 472.9 ± 17.30, *P* < 0.05). (c) Western blot results showed that NR treatment significantly reduced the Caspase-1/Caspase-1 pro and cleaved GSDMD expression in TAC mice (*P* < 0.05). Furthermore, NR markedly reduced the NLRP3 expression in the TAC+NR group (*P* < 0.05). There was no significant difference in ASC expression (*P* > 0.05). NR: nicotinamide riboside. ^∗^*P* < 0.05 vs. sham group; ^#^*P* < 0.05 vs. TAC group.

The results of ELISA and Western blot measurements of inflammatory cytokines are shown in Figure 3. Levels of inflammatory factors, IL-1*β* and TNF-*α*, were increased in the myocardial tissue of the TAC group (IL-1*β*: TAC: 267.58 ± 10.23 vs. sham: 195.32 ± 6.23; *P* < 0.05; TNF-*α*: TAC: 472.9 ± 17.30 vs. sham: 312.2 ± 18.11; *P* < 0.05), and NR treatment significantly reduced these levels (IL-1*β*: TAC+NR: 233.17 ± 7.12 vs. TAC: 267.58 ± 10.23; *P* < 0.05; TNF-*α*: TAC+NR: 366.9 ± 13.40 vs. TAC: 472.9 ± 17.30; *P* < 0.05). Western blot results (Figure 3(c)) showed that the ratio of Caspase-1/Caspase-1 pro in the myocardium of the TAC group increased (*P* < 0.05), along with the expression of cleaved GSDMD and NLRP3 protein. NR treatment reduced the expression of NLRP3 and cleaved GSDMD, along with Caspase-1 activation in the TAC+NR group (*P* < 0.05). These results demonstrate the inhibitory effects of NR on the inflammasome activation induced by TAC.

### 3.4. NR Alleviated TAC-Induced Oxidative Stress

**Figure 4:**
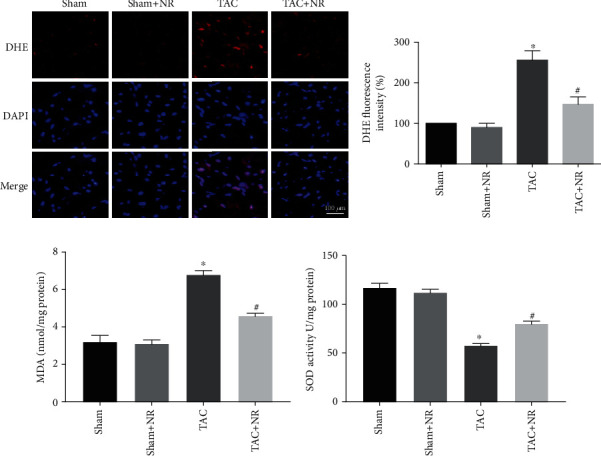
NR alleviates TAC-induced oxidative stress. (a) Myocardial DHE staining images. Scale bar: 100 *μ*m. (b) DHE fluorescence intensity revealed that NR treatment markedly reduced the augmentation of superoxide generation in the TAC+NR group (*P* < 0.05). (c) NR treatment markedly attenuated the increased MDA content level in TAC mice (*P* < 0.05). (d) NR restored the impaired SOD activity in TAC mice (*P* < 0.05). ^∗^*P* < 0.05 vs. sham group; ^#^*P* < 0.05 vs. TAC group.

Mitochondrial ROS production was measured via DHE staining of myocardial tissue. Figures 4(a) and 4(b) show that the DHE fluorescence intensity was increased in the TAC myocardial tissue (*P* < 0.05), an effect which was reduced by NR treatment (*P* < 0.05). In addition, the myocardial MDA level was elevated in the TAC group compared with the sham, suggesting severe oxidative stress (*P* < 0.05). NR treatment reduced MDA levels (*P* < 0.05, Figure 4(c)). Furthermore, TAC also impaired the activity of the myocardial antioxidant enzyme, SOD, the activity of which was restored by NR treatment (*P* < 0.05, Figure 4(d)).

### 3.5. NR Regulates NLRP3 Inflammasome Activation through the NAD^+^-Sirtuin3-MnSOD Axis

**Figure 5:**
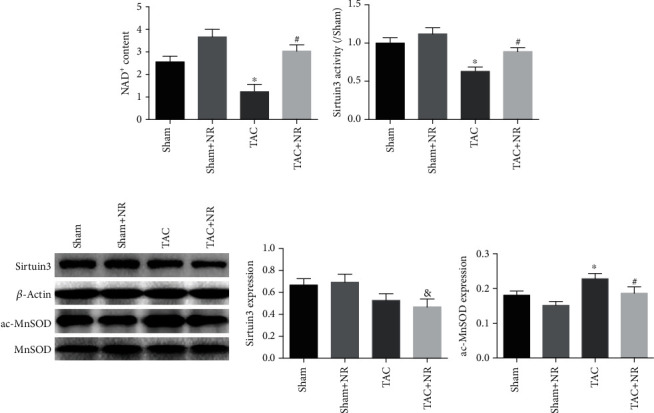
NR regulates the NAD^+^-Sirtuin-MnSOD axis. (a) The NAD^+^ content was reduced in TAC grouped myocardium, while NR significantly elevated the NAD^+^ content in the TAC+NR group (*P* < 0.05). (b) NR treatment can significantly increase the activity of Sirtuin3 protein in the TAC+NR group (*P* < 0.05). (c) Western blot results show decreased Sirtuin3 expression in the TAC and TAC+NR groups (*P* < 0.05). The acMOD protein level in the myocardial tissue of mice was significantly increased in the TAC group, while NR treatment can significantly reduce the acMOD protein level (*P* < 0.05). ^∗^*P* < 0.05 vs. sham group; ^#^*P* < 0.05 vs. TAC group; ^&^*P* < 0.05 vs. sham+NR group.

The therapeutic effectiveness of NR is dependent on the NAD^+^ pool.  Figure 5(a) shows that NAD^+^ content was reduced in the myocardial tissue of the TAC group (*P* < 0.05), an effect which was ameliorated by NR treatment (*P* < 0.05). Moreover, the TAC-induced reduction of Sirtuin3 protein was also ameliorated by NR treatment (*P* < 0.05, Figure 5(b)). MnSOD is the regulatory substrate of Sirtuin3 and a key protein in the regulation of cellular oxidative stress.  Figure 5(c) shows that levels of ac-MnSOD protein increased after TAC treatment, an effect which was partially reversed by NR treatment (*P* < 0.05). These changes are consistent with the altered levels of Sirtuin3 activity.

## 4. Discussion

In the present study, we have demonstrated that NLRP3 inflammasome activation, along with accompanying secretion of cytokines, contributed to the progression of cardiac hypertrophy in a TAC mouse model. Furthermore, our data established the therapeutic potential of NR, an NAD^+^ booster which can be administered orally through the diet. The underlying mechanism was revealed to involve the Sirtuin3-MnSOD signaling pathway and its inhibition of the NLRP3 inflammasome.

Individuals with cardiac hypertrophy are vulnerable to heart failure and arrhythmia as a result of pathological cardiac remodeling [12]. Hence, there is an urgent need to investigate the mechanistic causes of hypertrophic progression. Previous studies have implicated inflammatory processes in the pathophysiology of cardiac hypertrophy, and the identification of such mechanisms exposes potential targets for pharmacological modulation [13, 14]. NLRP3 is a pattern recognition receptor (PRR) which allows the cell to respond to danger signals. Activated NLRP3 interacts with adapter apoptosis-associated speck-like protein containing a C-terminal caspase recruitment domain (ASC) to form the NLRP3 inflammasome which activates Caspase-1 and results in the production of proinflammatory cytokines [15]. The current study establishes the presence of excessive NLRP3 inflammasome activation in TAC-induced cardiac hypertrophy and dysfunction. Inhibition of the NLRP3 inflammatory response had a beneficial impact on the hypertrophic heart. The findings of previous work have also suggested that selective NLRP3 inflammasome inhibition has a cardioprotective effect, reducing the risk of heart failure or other cardiovascular diseases [16, 17]. However, there is a certain degree of controversy surrounding these conclusions. Li et al. reported opposing findings that NLRP3 expression was downregulated during the hypertrophic process and the deficiency of NLRP3 exacerbated pressure overload-induced cardiac remodeling [18]. However, the apparent discrepancy between these results and the conclusions of the current study may be explained by the effect of NLRP3 protein on downregulating Toll-like receptor (TLR) 4 and does not take into account the more wide-ranging role of the complete NLRP3 inflammasome. The balance of opinion is predominantly in favor of the detrimental effect that the NLRP3 inflammasome contributes to the pathological process of cardiovascular disease and acknowledges that the NLRP3 inflammasome is a promising therapeutic target [19, 20].

NAD^+^ is an abundant molecule which is ubiquitous in its participation in biological processes [21]. In the last decade, there has been renewed interest in NAD^+^ as a result of its association with the Sirtuins (Sirtuin1–7), a family of NAD-dependent protein deacylases [22]. Levels of NAD^+^ are considered to decrease with age, and pathological condition and NAD^+^ supplementation have been suggested as a therapy for cardiovascular diseases [23]. The findings of the current study indicate that levels of NAD^+^ were reduced in the TAC mouse model. Supplementation with NR, a pharmacological NAD^+^ precursor, contributed to the restoration of heart function and remodeling. A previous clinical study has reported that a single oral dose of 1000 mg NR raised the blood NAD^+^ level by 2.7-fold, demonstrating the feasibility of NAD^+^ supplementation for the human patient [23]. More recently, Zhou et al. have reported attenuated proinflammatory activation of heart failure in patients given oral NR to augment NAD levels [24]. Collectively, these studies indicate great potential for clinical NAD^+^ repletion in the treatment of cardiovascular disorders. Further research is necessary to clarify the therapeutic potential of NAD^+^ booster treatment to ameliorate cardiac hypertrophy in patients.

The current study also explored the relationship between NAD^+^ supplementation and NLRP3 inflammasome activation in hypertrophic hearts. Generation of mitochondrial ROS is considered a key to NLRP3 inflammasome activation [25, 26]. Thus, we considered the possibility that regulation of oxidative stress might favorably orchestrate NLRP3 inflammatory responses. We found that NR supplementation attenuated TAC-induced myocardial oxidative stress, and this showed a consistent relationship with NLRP3 inflammasome activation. These findings strongly suggest that the inhibitory effect of NR on the NLRP3 inflammasome could be attributed to mitochondrial ROS production.

Previous studies have revealed reduced Sirtuin3 expression and elevated lysine acetylation of mitochondrial proteins in models of hypertensive heart failure, indicating an association of impaired Sirtuin3 activity with pathological cardiac remodeling [27]. Our findings demonstrated that boosting NAD^+^ levels through NR supplementation caused elevated Sirtuin3 activity and deacetylation of MnSOD and alleviated TAC-induced myocardial oxidative stress. These findings from the current study are in agreement with preclinical data reported by Lee et al. who found that normalization of the NADH/NAD^+^ imbalance attenuated mitochondrial protein hyperacetylation in heart failure models [9]. The current study identified that the beneficial effect of Sirtuin3 was mediated by Mn superoxide dismutase (MnSOD), a superoxide scavenger, with reduced superoxide production resulting in attenuation of oxidative stress. However, a previous study by Diguet et al. observed that dietary NR supplementation attenuates the development of HF in mice without an impact on global cardiac protein deacetylation, and they observed robustly increased acetylation levels of FOXO1 and p53 transcription factors. By contrast, we have presented evidence that NR causes elevated myocardial Sirtuin3 activity and decreased acetylation level of MnSOD [28]. There are a number of acetylases and deacetylases, some of which are NAD^+^-dependent while others are NAD^+^-independent. Further preclinical and clinical investigations of the impact of augmented NAD^+^ on protein acetylation are warranted.

The present study confined the analysis to the activity of Sirtuin3, a member of the larger family of Sirtuin proteins. Sirtuins1-7 are major downstream mediators of the NAD^+^ regulation of biological processes [22]. There have been several studies which have reported the NAD^+^-dependent activation of Sirtuin1 and Sirtuin6 following supplementation with NR [29–31]. It is conceivable that other members of the Sirtuin family are also targets for NR supplementation in the context of the TAC model. Further investigations are required to better understand the effects and underlying mechanisms involved in NAD^+^ booster treatment.

## 5. Conclusions

Taken all together, the findings of the present study demonstrate the utility of NR as a booster for NAD^+^ levels in attenuating cardiac hypertrophy induced by pressure overload. We have revealed a mechanism associated with reduced oxidative stress and inhibition of NLRP3 inflammasome activation via the Sirtuin3-MnSOD signaling pathway. We conclude that NR, an NAD^+^ booster that can be given through the diet, has great promise as a novel therapeutic intervention for cardiac hypertrophy.

## Figures and Tables

**Table 1:** Primer sequences for real-time PCR.

	Forward primer	Reverse primer
ANP	GCTTCCAGGCCATATTGGAGCA	TCTCTCAGAGGTGGGTTGACCT
BNP	ATGGATCTCCTGAAGGTGCTGT	GCAGCTTGAGATATGTGTCACC
GAPDH	GGCACAGTCAAGGCTGAGAATG	ATGGTGGTGAAGACGCCAGTA

## Data Availability

Data are available on request.
